# Supplementary material for: The interactome of intact mitochondria by cross-linking mass spectrometry provides evidence for coexisting respiratory supercomplexes
Source: Mol Cell Proteomics. 2017 Dec 8;17(2):216–32. doi: 10.1074/mcp.RA117.000470 (PMC5795388; doi:10.1074/mcp.RA117.000470)
Supplement: Supplemental Data [file supp_RA117.000470_133922_0_supp_23978_fzffwf.zip › spectra_annotation/mito_DR_spectra_annotation/50-1-2-1-1-1.pdf]

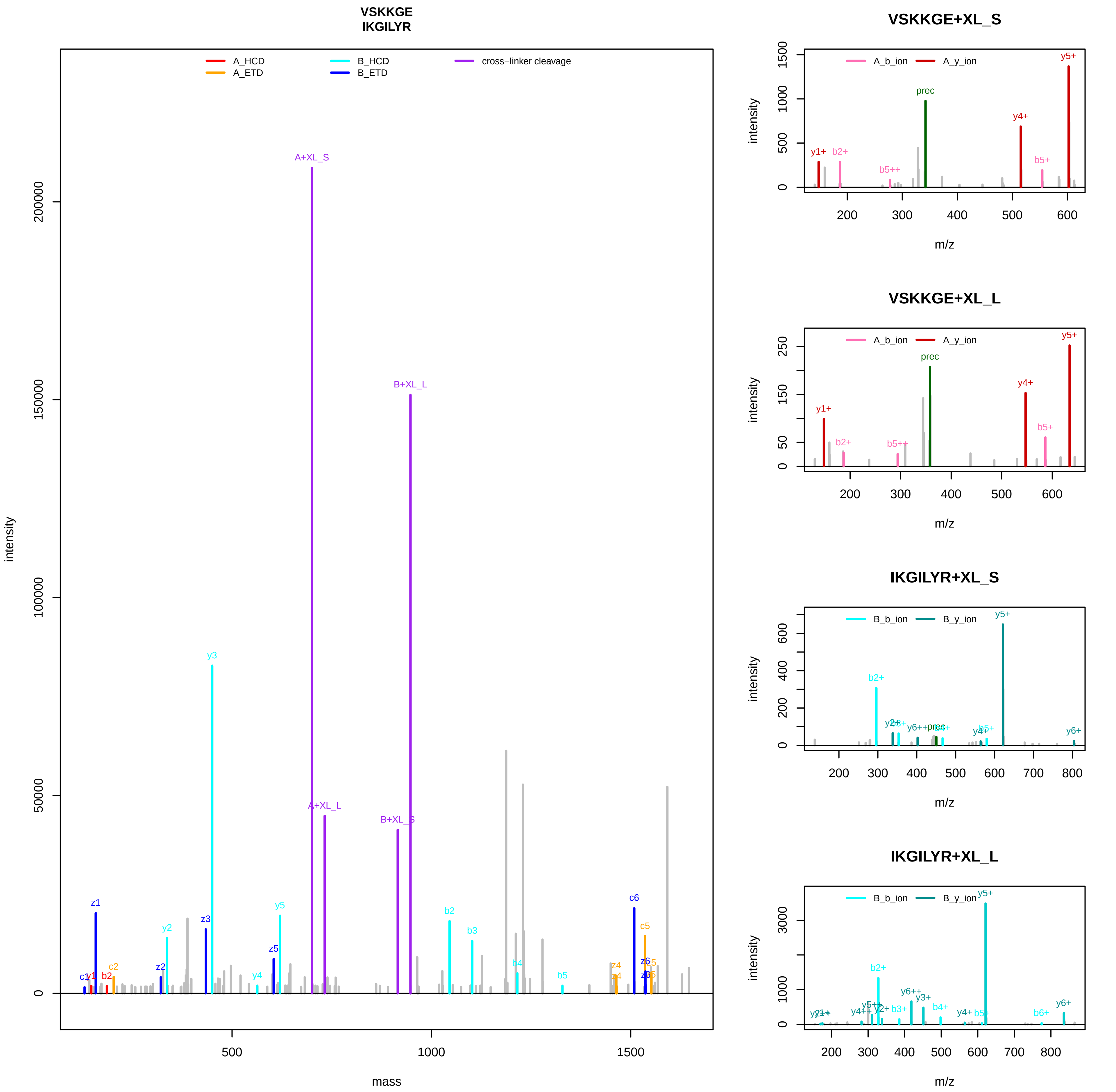
VSKKGE
IKGILYR
A_HCD
A_ETD
B_HCD
B_ETD
cross−linker cleavage
intensity
mass
A+XL_S
B+XL_L
A+XL_L
B+XL_S
y3
z1
c1
y1
b2
c2
z2
y2
z3
y4
z5
y5
b2
b3
b4
b5
c6
c5
z4
z6
0
50000
100000
150000
200000
500
1000
1500
VSKKGE+XL_S
VSKKGE+XL_L
IKGILYR+XL_S
IKGILYR+XL_L
A_b_ion
A_y_ion
B_b_ion
B_y_ion
prec
m/z
y1+
b2+
b5++
y4+
b5+
y5+
y2+
y6++
y6+
b3+
b4+
y3+
y4++
y5++
b6+
